# Efficacy and safety of olaparib combined with abiraterone in patients with metastatic castration-resistant prostate cancer: a systematic review and meta-analysis of randomized controlled trials

**DOI:** 10.3389/fonc.2023.1265276

**Published:** 2023-10-06

**Authors:** Zhanyang Luo, Bukun Zhu, Hong Xu, Lixin Chen, Xiaoyun Song, Yu Wang, Rui Wang, Jinzhou Zheng, Yunhua Qiu, Jianfeng Yang, Youyang Shi

**Affiliations:** ^1^Longhua Hospital, Shanghai University of Traditional Chinese Medicine, Shanghai, China; ^2^Affiliated Hospital of Youjiang Medical University for Nationalities, Baise, China

**Keywords:** olaparib, abiraterone, metastatic castration-resistant prostate cancer (mCRPC), efficacy, safety, meta-analysis

## Abstract

**Background:**

Olaparib has been proven for the treatment of metastatic castration-resistant prostate cancer (mCRPC). This meta-analysis aims to comprehensively evaluate the efficacy and safety of the combination of olaparib and abiraterone in patients with mCRPC.

**Methods:**

The literature in PubMed, Embase, and Cochrane Library up until April 27, 2023, was systematically searched. In the studies included in this meta-analysis, olaparib combined with abiraterone was compared with abiraterone combined with placebo.

**Results:**

Two randomized controlled trials involving a total of 938 patients were included. Analysis indicated that olaparib combined with abiraterone significantly prolonged radiographic progression-free survival (rPFS: relative risk [RR] 0.66, 95% confidence interval [CI] 0.55–0.79), time to secondary progression or death (PFS2: hazard ratio [HR] 0.72, 95% CI 0.56–0.93), time to first subsequent therapy or death (TFST: HR 0.75, 95% CI 0.63–0.89), time to second subsequent therapy or death (TSST: HR 0.73, 95% CI 0.58–0.93), and confirmed prostate-specific antigen (PSA) response (RR 1.14, 95% CI 1.05–1.24). However, no statistically significant differences were found in the overall survival (OS: HR 0.87 95% CI 0.70–1.09), objective response rate (ORR: RR 0.97, 95% CI 0.70–1.33), and incidence of total adverse events (RR 1.07, 95% CI 0.94–1.22). A notable detail that the combination of olaparib and abiraterone was associated with an increased incidence of high-grade anemia (RR 7.47, 95% CI 1.36–40.88).

**Conclusion:**

Olaparib combined with abiraterone is effective for patients with mCRPC. However, combination therapy has treatment-related adverse events compared with monotherapy, and this could be improved in future treatment management.

**Systematic review registration:**

https://www.crd.york.ac.uk/PROSPERO/, identifier CRD42023432287.

## 1 Introduction

Prostate cancer is the second most common cancer in men, comprising 14.1% of all cases and accounting for 6.8% of all cancer-related mortality in 2020 worldwide (1). Despite survival and quality of life having greatly improved in patients using next-generation hormonal agents (NHA), chemotherapy or radiotherapy, metastatic castration-resistant prostate cancer (mCRPC) remains lethal and has poor prognosis (2). Approximately 20%–30% (3–5) of patients with mCRPC exhibit alterations in homologous recombination repair (HRR), specifically *BRCA1* and *BRCA2*. Moreover, HRR gene aberrations in patients with mCRPC are correlated with a poor prognosis, which renders them susceptible to poly(ADP-ribose) polymerase inhibitors (PARPis) (6).

Olaparib (Lynparza) was approved by the US Food and Drug Administration for the treatment of mCRPC harboring HRR deficiency after patients used next generation hormonal agents (7). In the phase III PROfound study (NCT02987543), olaparib monotherapy showed clinical improvement in radiographic progression-free survival (rPFS) and overall survival (OS) for patients with mCRPC who had a mutation in BRCA1, BRCA2, or ATM (8). Abiraterone acetate (hereafter abiraterone), as a selective and irreversible inhibitor of CYP17, effectively impedes androgen biosynthesis (9). Preclinical studies suggested that PARPi combined with NHA may be a novel antitumor therapy (10, 11). These studies reported that NHA can result in HRR deficiency by inhibiting transcription in some HRR genes, leading to sensitivity to PARPi of mCRPC.

Previous evidence from meta-analyses focused on assessing the efficacy of NHA and docetaxel (12) or PARPi (13–15). However, the systematic review of evidence supporting the use of combination of olaparib and abiraterone is limited and unclear. Hence, the present study aimed to comprehensively evaluate the efficacy and safety of the combination of olaparib and abiraterone in patients with mCRPC by pooling new data from two randomized controlled trials. Understanding the role of the above combination therapy in patients with mCRPC may enhance clinical decision-making.

## 2 Materials and methods

This meta-analysis was carried out on the basis of the guideline of Preferred Reporting Items for Systematic Reviews and Meta-Analyses (PRISMA) (16). The protocol was registered with the International Prospective Register of Systematic Reviews (PROSPERO, CRD42023432287).

### 2.1 Data sources and search strategy

The literature in PubMed, Embase, and Cochrane Library up until April 27, 2023, was systematically searched using the following term combinations: (“Olaparib or AZD2281 or KU0059436”) and (“prostatic neoplasms or prostatic carcinoma or prostate cancer”) and (“randomized controlled trial”). The search strategies for the three databases are fully provided in **Supplementary Table 1**. Besides, potential articles were manually searched in related studies and reviews. The eligibility of titles and abstracts in all articles was independently assessed by two reviewers (ZL and BZ).

### 2.2 Inclusion and exclusion criteria

On the basis of PICOS criteria, two reviewers independently assessed the studies by the initial literature search, subsequently incorporating the studies that met the eligibility criteria. The studies included in this meta-analysis met the following criteria:

Participants: must be diagnosed with mCRPC and at least 18 years of age.
Intervention: combination of olaparib and abiraterone.
Comparator: placebo or other active drugs.
Outcomes: radiological progression free survival (rPFS), overall survival (OS), time to first subsequent therapy or death (TFST), time to second subsequent anti-cancer therapy or death (TSST), time to secondary progression or death (PFS2), confirmed prostate-specific antigen (PSA) response, objective response rate (ORR), and adverse events.
Study design: the included studies had a phase II or III randomized control trial (RCT) design.

The exclusion criteria were as follows: phase I trials, letters, comments, reviews, or meta-analyses; studies without sufficient data and proper control drugs; and repeat publications.

When disagreements arose, a third reviewer was involved and evaluated all the eligible articles until a consensus was reached.

### 2.3 Data extraction and risk of bias assessment

In accordance with the Cochrane Handbook guidelines, two investigators (YQ and JY) independently assessed and extracted the proper data including: first author, publication year, NCT number, inclusion criteria, phase of study, intervention and control group, median follow-up, and survival endpoints.

The ROB of the included RCTs was assessed using version 1.0 of the Cochrane Handbook’s ROB tool, which involves five domains: random sequence generation, allocation concealment, blinding, incomplete outcome data, and selective reporting. The ROB of each included study was assessed independently by two reviewers (ZL and YS). Each domain was classified as having a high, low, or unclear ROB.

### 2.4 Definition of outcomes

The primary outcomes were rPFS and OS. The secondary outcomes included TFST, TSST, PFS2, confirmed PSA response, ORR, and adverse events.

rPFS is defined as the time from randomization to radiological progression (assessed by the investigator per the Response Evaluation Criteria in Solid Tumors [RECIST] 1.1 for soft tissue or the Prostate Cancer Working Group-3 [PCWG3] criteria for bone) or death from any cause (17).

OS is defined as the time from randomization to death from any cause (18).

TFST and TSST are defined as the time from randomization to the earlier of the first subsequent or the second subsequent anti-cancer therapy start date following study treatment discontinuation, or death, respectively.

PFS2 is defined as objective radiological progression by RECIST 1.1 for soft tissue, symptomatic progression, a rise in PSA level, or death in the absence of overall progression (19).

Confirmed PSA response is defined as a reduction in PSA level of 50% or more on two consecutive occasions at least 4 weeks apart compared with baseline (20).

ORR is defined as the percentage of patients with a complete response or partial response in soft tissue disease and a bone scan status of non-progressive disease (21).

The adverse events from olaparib combined with abiraterone and the control assessed in this article included total adverse events, grade 3 or worse adverse events, anemia, fatigue or asthenia, nausea, and diarrhea.

### 2.5 Data synthesis and statistical analysis

Statistical analysis was performed using Stata software version 12.0. For survival outcomes, the hazard Ratios (HRs) and 95% confidence intervals (CIs) for rPFS, OS, TFST, TSST, and PFS2 were extracted from each included article. Pooled HRs were obtained using the generic inverse of variance method with a random-effect model (22). When an HR < 1, it implied that the related endpoints of olaparib combined with abiraterone were associated with a lower risk than the control group. For dichotomous variables (i.e., ORR, confirmed PSA response and adverse events), relative risk (RR) was used to assess the differences between combination therapy and control interventions. The random-effect model was used to analyze all quantitative data. The results were analyzed and presented in forest plot. I^2^ statistic and the Cochrane Q statistic were used to assess between-study heterogeneity. A value of I^2^ greater than 50% and a *p*-value < 0.10 were considered indicative of a substantial level of heterogeneity (23). A *p*-value < 0.05 was considered statistically significant.

## 3 Results

### 3.1 Study selection and characteristics

**Figure 1:**
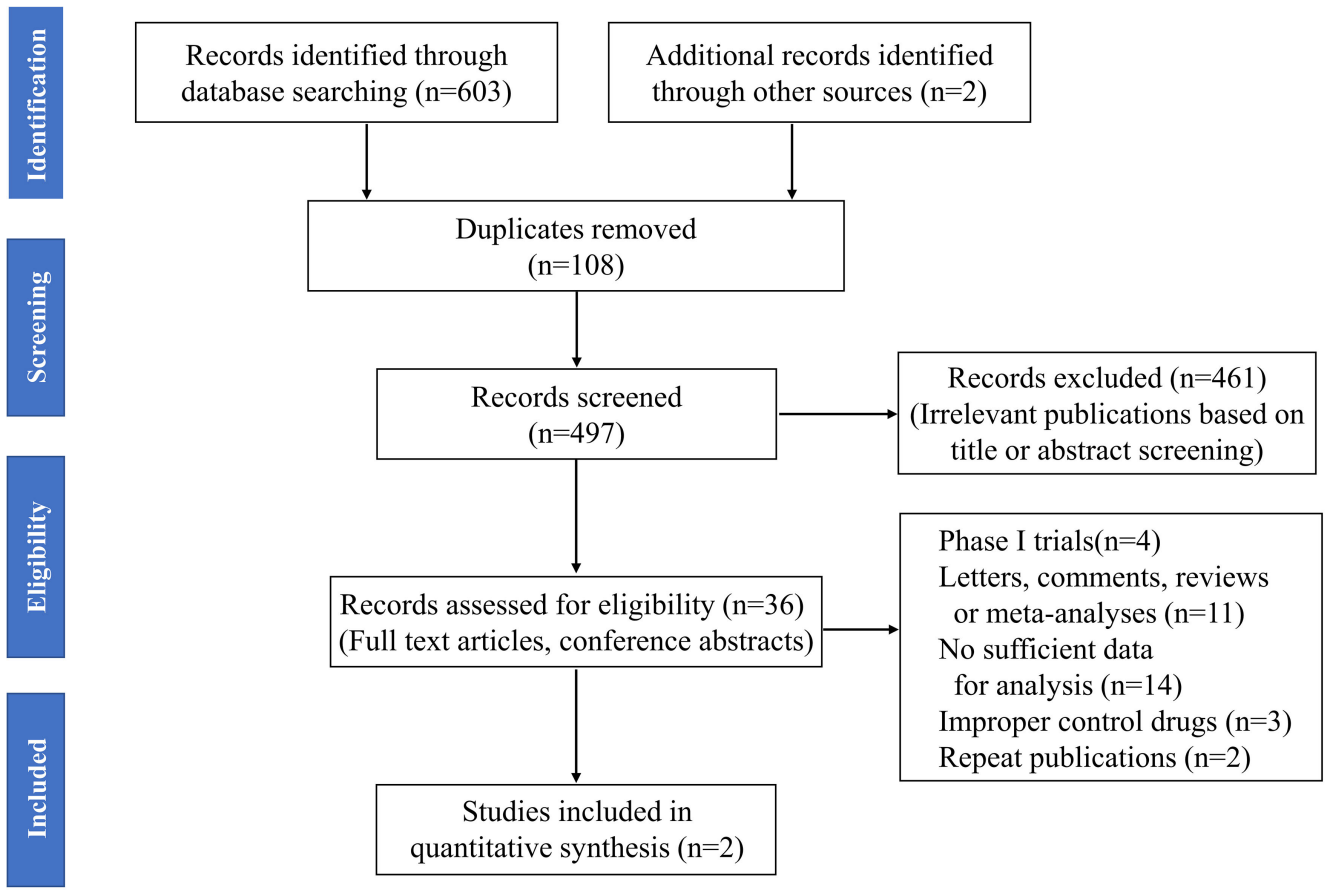
Flowchart of study screening in this meta-analysis.

The results of all records identified in the search are shown in a flow diagram (**Figure 1**). A total of 603 studies were identified through the systematic literature search. Two potential records were also identified from the references of the reviews by hand searching. After 108 duplicate articles and 461 irrelevant articles were excluded on the basis of title or abstract screening, a total of 36 full-text articles and conference abstracts were considered potentially eligible for this review. Subsequently, 34 studies were excluded, including four with phase I trials; 11 with letters, comments, reviews, or meta-analyses; 14 with insufficient data; three with improper control drugs; and two with repeat publications. Finally, only 2 RCTs were included for assessment in this meta-analysis (24, 25).

**Figure 2:**
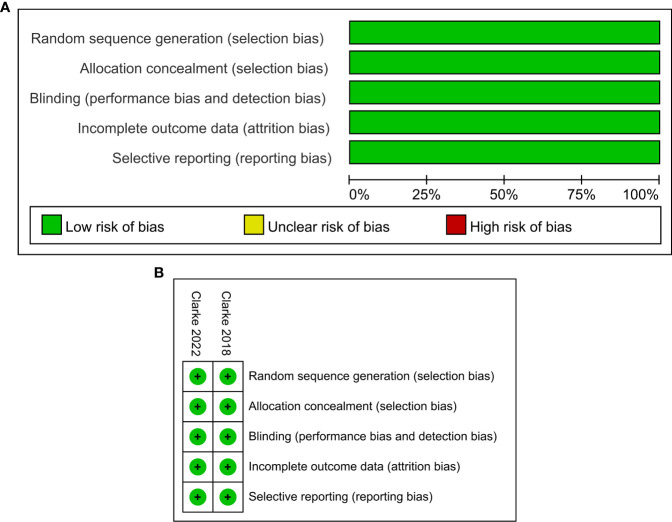
Risk of bias graph: reviewers’ judgments about each risk of bias item presented as percentages across all included studies **(A)**. Risk of bias summary: reviewers’ judgments about each risk of bias item for each included study according to the Cochrane Collaboration’s “Risk of Bias” tool, the green circle with “plus” sign representing low risk of bias, the yellow circle with “question mark” sign representing unclear risk of bias and the red circle with “minus” sign represents high risk of bias **(B)**.

The characteristics of the included studies in this meta-analysis are summarized in **Table 1**. Trials involving a total of 938 patients, including one phase II (24) and one phase III (25) trials, were conducted in Europe, America, Asia, and Oceania. All participants had histologically confirmed mCRPC, which included 470 men receiving olaparib combined with abiraterone and 468 men receiving abiraterone combined with placebo. Olaparib was administered orally at a dose of 300 mg two times daily, and abiraterone was administered orally at a dose of 1000 mg per day. More details about the characteristics of patients at baseline are shown in **Supplementary Table 2**. The ROB graph (**Figure 2**) indicated that the ROB was low across all domains.

**Table 1:** Characteristics of included randomized controlled trials.

First author year	NCT number	Inclusion criteria	Phase	Intervention (N)	Control (N)	Median follow-up (months)	Survival endpoints
**Clarke** **2018 **(24)	NCT01972217	Patients had mCRPC, and had previously received docetaxel and up to one additional line of previous chemotherapy.	II	Olaparib +abiraterone (71)	Abiraterone +placebo (71)	Intervention: 15.9 Control: 24.5	rPFS, OS, PFS2, TFST, TSST, ORR, PSA response, adverse events, *etc.*
**Clarke** **2022 **(25)	NCT03732820	Patients had histologically or cytologically confirmed prostate cancer with at least one documented metastatic lesion.	III	Olaparib +abiraterone (399)	Abiraterone +placebo (397)	Intervention: 19.3 Control: 19.4	rPFS, OS, ORR, PSA, time to PSA progression, adverse events, *etc.*

NCT, ClinicalTrials.gov number; mCRPC, metastatic Castration-resistant prostate cancer; rPFS, radiologic Progression-free survival; OS, overall survival; TFST, time to first subsequent therapy or death; TSST, time to second subsequent anti-cancer therapy or death; ORR, objective response rate; PSA, prostate-specific antigen.

### 3.2 Results of the meta-analysis

#### 3.2.1 Primary outcomes

##### 3.2.1.1 Radiologic progression-free survival

**Figure 3:**
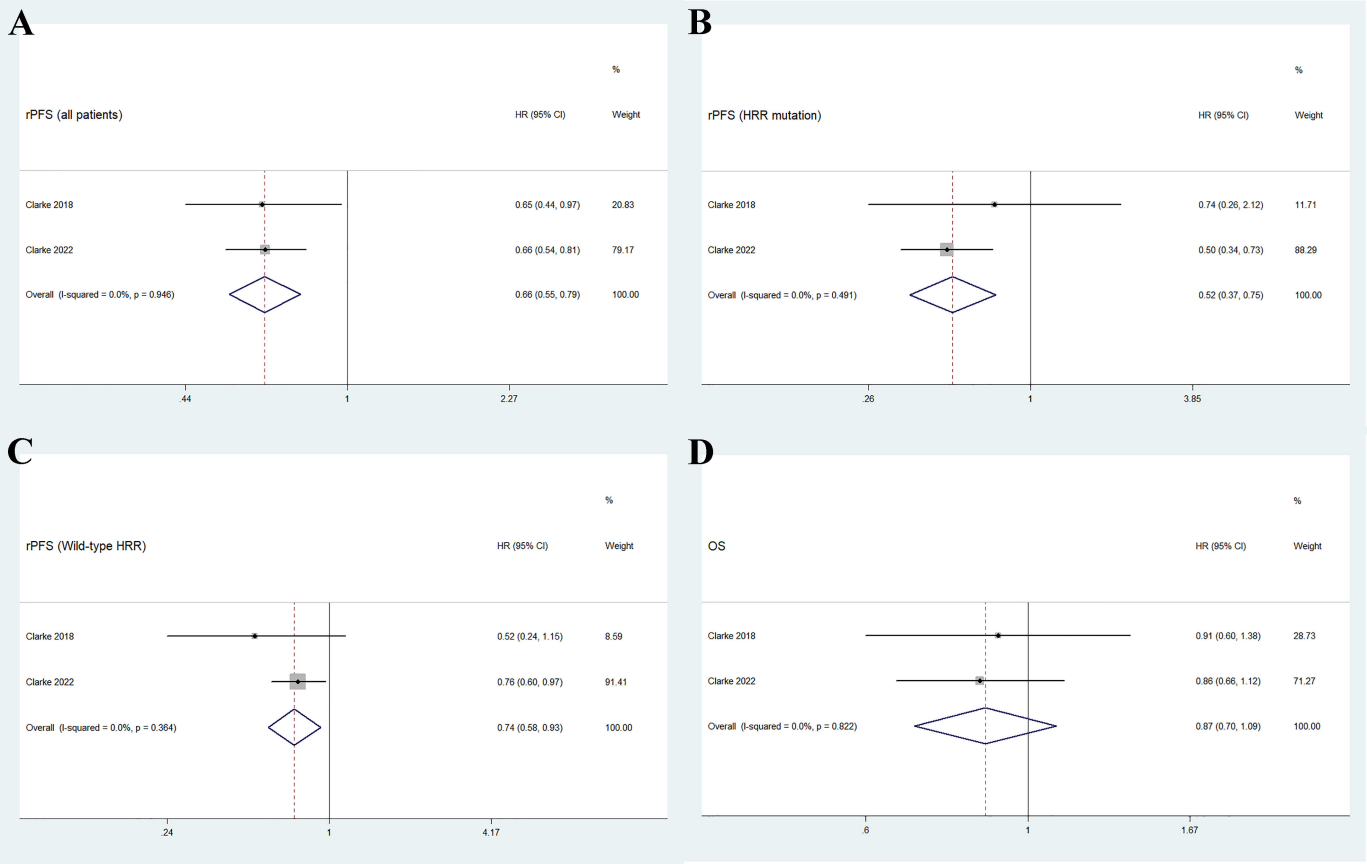
Forest plot of randomized controlled trials on olaparib combination therapy for primary outcomes: rPFS **(A)**, rPFS in HRR mutation subgroup **(B)**, rPFS in wild-type HRR subgroup **(C)**, and OS **(D)**.

As shown in **Figure 3A**, the pooled HR for rPFS in the total patient population comparing olaparib combined with abiraterone versus abiraterone combined with placebo was 0.66 (95% CI 0.55–0.79). The use of olaparib in combination with abiraterone was significantly associated with improved rPFS in patients with mCRPC (n = 938, *p* < 0.01), and no significant heterogeneity was found between the studies (I^2 =^ 0%, *p* = 0.946). Moreover, the subgroup analysis that considered HRR mutation status to HRR mutation/wild-type showed that the olaparib combination group had favorable rPFS benefits (HRR mutation: n = 247, HR 0.52, 95% CI 0.37–0.75; wild-type HRR: n = 587, HR 0.74, 95% CI 0.58–0.93; *p* < 0.01), as illustrated in **Figures 3B, C**. No significant heterogeneity existed across the studies in neither the HRR mutation group (I^2 =^ 0%, *p* = 0.491) nor the wild-type HRR group (I^2 =^ 0%, *p* = 0.364).

##### 3.2.1.2 Overall survival

Two studies reported the outcome of OS in patients with mCRPC, with a total of 938 individuals. **Figure 3D** shows that the pooled HR for OS comparing olaparib combined with abiraterone and abiraterone combined placebo was 0.87 (95% CI 0.70–1.09, *p* = 0.237), indicating no significant differences between the olaparib combination group and the control group, and no significant heterogeneity (I^2 =^ 0%, *p* = 0.822).

#### 3.2.2 Secondary outcomes

##### 3.2.2.1 Time to secondary progression or death, first and second subsequent therapy or death

**Figure 4:**
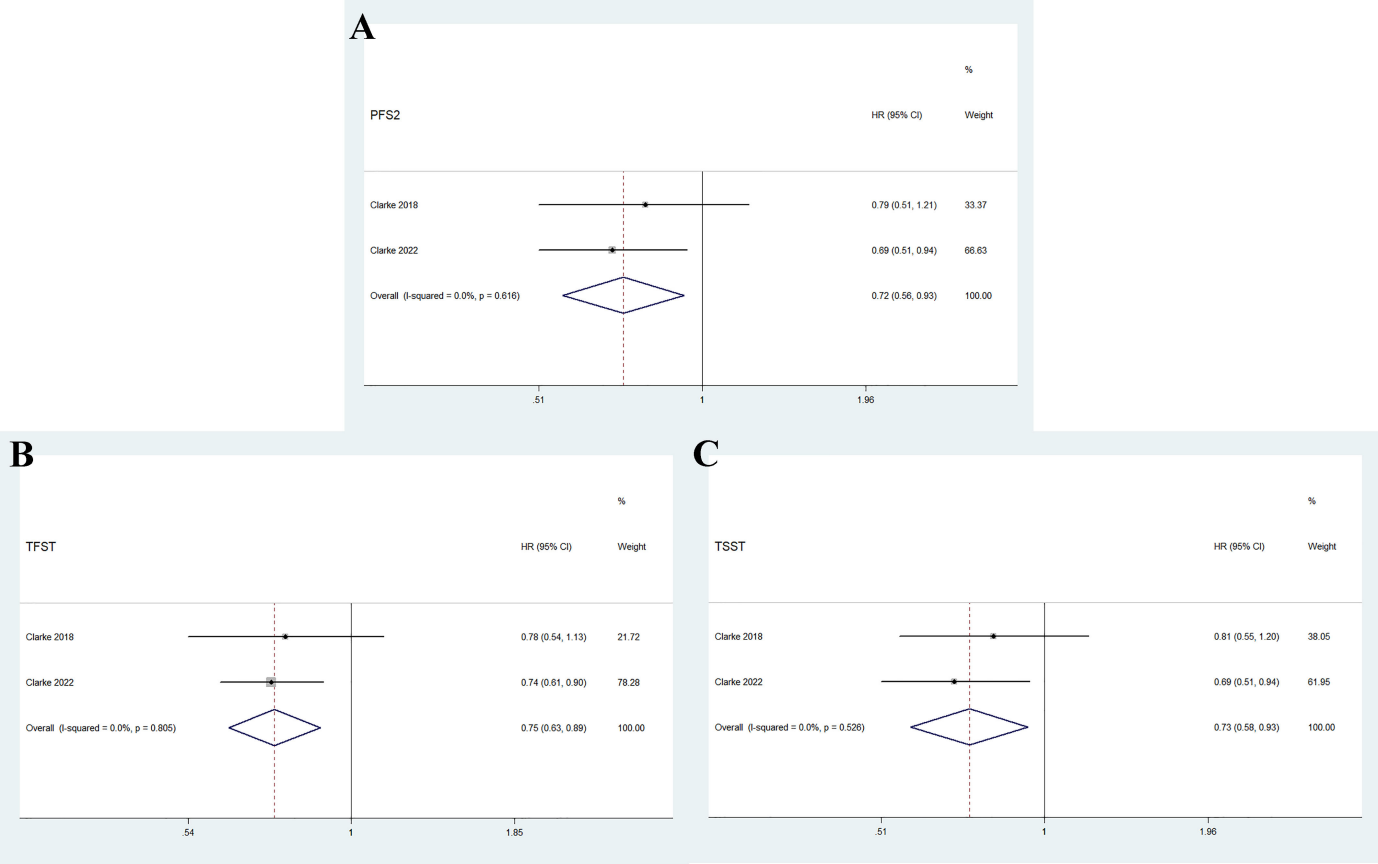
Forest plot of randomized controlled trials on olaparib combination therapy for secondary outcomes: PFS2 **(A)**, TFST **(B)**, and TSST **(C)**.

As shown in **Figure 4**, the pooled results indicate that olaparib combined with abiraterone significantly prolonged PFS2 (HR 0.72, 95% CI 0.56–0.93, *p* = 0.01), TFST (HR 0.75, 95% CI 0.63-0.89, *p* = 0.001), and TSST (HR 0.73, 95% CI 0.58–0.93, *p* = 0.012) compared with abiraterone plus placebo. No significant heterogeneity was observed between the olaparib combination group and the abiraterone group (I^2 =^ 0%, *p* > 0.10).

##### 3.2.2.2 Objective response rate and confirmed prostate-specific antigen response

**Figure 5:**
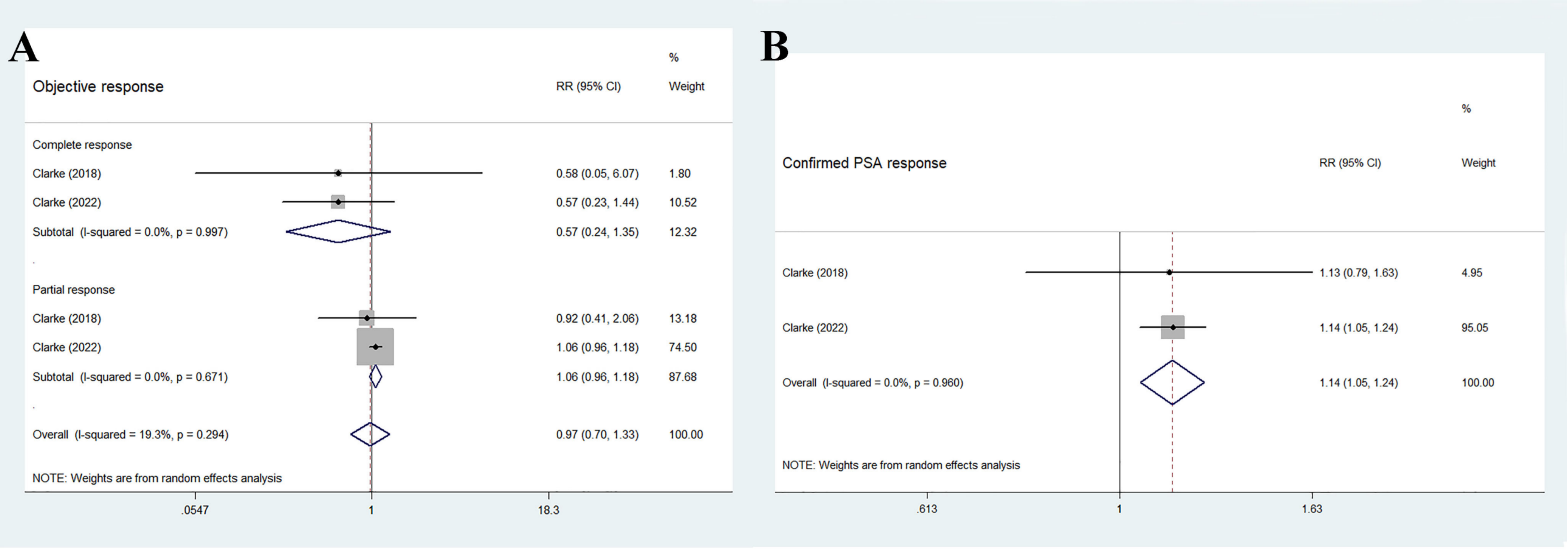
Forest plot of randomized controlled trials on olaparib combination therapy for secondary outcomes: ORR **(A)** and confirmed PSA response **(B)**.

The two RCTs reported the ORR, including a total of 194 individuals in the olaparib combination group and 198 individuals in the control group. The overall results showed no significant difference between the two groups (RR 0.97, 95% CI 0.70–1.33, *p* = 0.838), and no significant heterogeneity (I^2 =^ 19.3%, *p* = 0.294), as shown in **Figure 5A**. Further subgroup analysis indicated that the two treatment groups had similar effects on complete response (RR 0.57, 95% CI 0.24–1.35, *p* = 0.203) and partial response (RR 1.06, 95% CI 0.96–1.18, *p* = 0.257). Furthermore, no significant inter-study heterogeneity was observed (complete response: I^2 =^ 0%, *p* = 0.997; partial response: I^2 =^ 0%, *p* = 0.671).

As shown in **Figure 5B**, the findings of the pooled data revealed that the olaparib combination therapy was superior in terms of confirmed PSA response (RR 1.14, 95% CI 1.05–1.24, *p* = 0.001), but no significant heterogeneity was observed between the two groups (I^2 =^ 0%, *p* = 0.96).

##### 3.2.2.3 Adverse events

**Figure 6:**
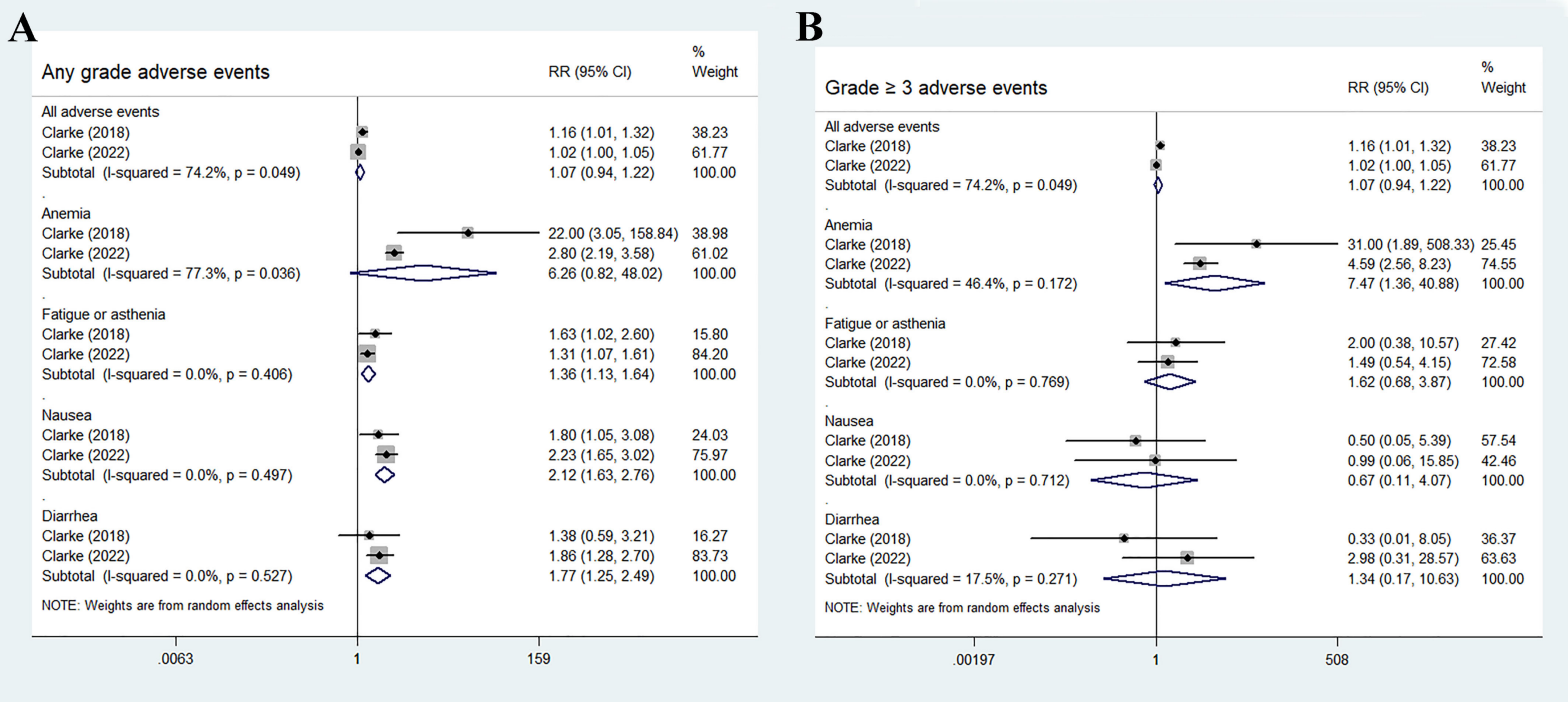
Forest plot of randomized controlled trials on olaparib combination therapy for secondary outcomes: any grades adverse events **(A)** and grade 3 or more severe adverse events **(B)**.

The most common adverse events in the olaparib and abiraterone groups were anemia, fatigue or asthenia, nausea, and diarrhea. No statistical differences were found in the incidence risk of total adverse events and anemia between the treatment groups and control groups (total adverse events: RR 1.07, 95% CI 0.94–1.22, *p* = 0.283; anemia: RR 6.26, 95% CI 0.82–48.02, *p* = 0.078). Fatigue or asthenia, nausea, and diarrhea were more common in the olaparib and abiraterone combination group than in the abiraterone group (fatigue or asthenia: RR 1.36, 95% CI 1.13–1.64, *p* = 0.001; nausea: RR 2.12, 95% CI 1.63–2.76, *p* < 0.01; and diarrhea: RR 1.77, 95% CI 1.25–2.49, *p* = 0.001), **Figure 6A**.

For grade 3 or more severe adverse events, the occurrence of anemia significantly increased in the olaparib and abiraterone group (RR 7.47, 95% CI 1.36–40.88, *p* = 0.02). However, no significant differences were observed in the occurrence of other adverse events between the two groups (all adverse events: RR 1.07, 95% CI 0.94–1.22, *p* = 0.283; fatigue or asthenia: RR 1.62, 95% CI 0.68–3.87, *p* = 0.28; nausea: RR 0.67, 95% CI 0.11–4.07, *p* = 0.663; and diarrhea: RR 1.34, 95% CI 0.17–10.63, *p* = 0.779), as shown in **Figure 6B**.

## 4 Discussion

### 4.1 Findings and interpretations

To the best of our knowledge, this study is the first meta-analysis pooling the data from two RCTs involving a total of 938 patients with mCRPC, and comparing the combination use of olaparib with abiraterone versus abiraterone monotherapy. A key finding of this systematic review is that the olaparib combination therapy offers a considerable rPFS benefit for patients with mCPPC compared with abiraterone monotherapy. Furthermore, patients who received olaparib experienced significant benefits in terms of PFS2, TFST, TSST, and confirmed PSA response compared with those who received abiraterone. Meanwhile, the olaparib combination therapy showed a similar effect on OS, ORR, and several adverse events compared with abiraterone monotherapy.

The primary objective of this review was to evaluate the rPFS of olaparib in combination with abiraterone for patients with mCRPC. The data indicated that this combination offers a substantial rPFS benefit for patients with mCRPC, regardless of HRR mutation status (HR 0.66; 95% CI, 0.55–0.79; *p* < 0.01). Consistent with previous studies (10), the findings of the present study validated the synergistic interaction between PPAR inhibitor (olaparib) and androgen receptor antagonist (abiraterone), regardless of HRR mutation status. Previous studies (26–28) evaluating castration and androgen deprivation in prostate cancer revealed a potential connection between the androgen pathway and the DNA damage response. Inhibition of androgen signaling seems to decrease the expression of HRR genes and impair the ability of cells to repair DNA double-strand breaks, leading to increased sensitivity to RARP inhibition. This evidence could potentially elucidate the rationale behind the enhanced effectiveness of olaparib combination therapy.

The evaluation of the efficacy of anti-tumor drugs in clinical trials often relies on assessing the clinical outcome of OS (18). OS is considered one of the most important measures as it provides unambiguous and unbiased results. Positive outcomes in OS can serve as confirmatory evidence that a particular drug has potential to prolong life. However, in the present study, the combination of olaparib and abiraterone failed to show a statistically prolonged OS in patients with mCRPC. This finding may be attributed to factors such as the limited sample size and baseline prognostic factors (age, ECOG status and PSA concentration). A retrospective analysis of 15 studies demonstrated a robust correlation between PFS2 and OS, suggesting that PFS2 can serve as a reliable measure of long-term clinical benefit in cases where OS assessment is not feasible (29). By contrast, the present study showed a significant benefit in PFS2 (HR 0.72, 95% CI 0.56–0.93, *p* < 0.05). One plausible explanation for the disparity observed between rPFS and OS could be the influence of crossover and post-progression therapies (30). In clinical practice, the need for immediate subsequent treatment following disease progression may not always be necessary, because the process of clinical decision-making should involve a comprehensive evaluation of the patient’s physical health, tumor status, and clinical symptoms. Consequently, the time to subsequent therapy may be more clinically meaningful than the time to progression alone for patients. The findings of the present study suggested that the combination of olaparib and abiraterone has potential to prolong TFST (HR 0.75, 95% CI 0.63–0.89, *p* < 0.01) and TSST (HR 0.73, 95% CI 0.58–0.93, *p* < 0.05).

In this study, the ORR in the olaparib combination and control groups were 53.1% and 44.9%, respectively, without statistical significance (RR = 1.18, 95% CI 0.97–1.44, *p* > 0.05). The subgroup analysis revealed similar results for complete and partial responses (*p* > 0.05). However, a higher proportion of patients in the combination therapy group achieved a confirmed PSA response rate of at least 50% (349 [74.3%] of 470 patients) compared with the monotherapy group (304 [65.0%] of 468, RR 1.14, 95% CI 1.05–1.24, *p* < 0.01). A phase I clinical trial reported that abiraterone achieved a decline in PSA concentration in 58% of patients with mCRPC (31). In the present study, olaparib and abiraterone demonstrated a good activity in reducing PSA levels.

In this study, the four most commonly reported adverse events were anemia, fatigue or asthenia, nausea, and diarrhea. Olaparib combination therapy resulted in higher incidences of high-grade anemia and any-grade adverse events (fatigue or asthenia, nausea, and diarrhea; *p*<0.05) than abiraterone monotherapy, whereas similar effects were observed in the other adverse events (*p*>0.05). In total, 43.7% of patients in the olaparib combination group experienced any-grade anemia (16.0% had grade 3 or higher), compared with 14.1% of patients in the control group (2.8% had grade 3 or higher). A recent meta-analysis of 29 trials found that anemia is the most frequently reported hematological toxicity associated with PARPi (such as olaparib, rucaparib, veliparib, niraparib and talazoparib) when compared with alternative treatment (32). However, the underlying cause of anemia resulting from PARPi remains poorly understood. Preclinical evidence suggested that PARP-2 plays a crucial role in erythroid differentiation, and its deletion can lead to extravascular hemolytic anemia. This finding revealed that PARPi may influence hematopoiesis, potentially explaining the observed hematological adverse events in these studies (33, 34). Recent evidence revealed that olaparib induced temporary macrocytic anemia. However, the contribution of potential deficiencies in the vitamin B12 or folic acid pathway to the development of this anemia remains uncertain (35). A notable detail that additional toxicities were frequently observed, unrelated to hematological effects. For patients with underlying disorders, conducting a comprehensive evaluation of the risks and benefits, and closely monitoring safety during the initial stages of treatment are crucial. Considering these factors, healthcare professionals should be well-informed about the potential adverse events, including fatigue, nausea, and diarrhea, that may occur in patients receiving PARPi. They should also utilize laboratory tests, physical examinations, and clinical judgment before and during the treatment period to ensure thorough monitoring.

### 4.2 Strengths and limitations

This meta-analysis possesses several notable strengths, including a comprehensive search across relevant databases and the inclusion of recent high-quality, well-designed, randomized, double-blinded trials. To our knowledge, this review is the first to systematically explore the efficacy and safety of olaparib and abiraterone combination therapy in patients with mCRPC. However, this systematic review had some unavoidable limitations. Firstly, the investigation of the effect of olaparib in combination with abiraterone on patients with different HRR mutation statuses is limited by the current lack of available phase II/III trials. Second, the inclusion of OS as a secondary endpoint in this study does not provide direct evidence of clinical benefit. Lastly, the number of studies (<10) included in the analysis is limited, and funnel plotting or Egger’s test was not conducted to assess the potential publication bias.

## 5 Conclusion

The analysis revealed that combination of olaparib and abiraterone significantly prolonged rPFS, PFS2, TFST, TSST and confirmed PSA response in patients with mCRPC. However, statistically significant differences were found in terms of OS and ORR. Regarding safety, no significant difference was observed in the incidence of adverse events between the olaparib combination group and the control group. The combination of olaparib and abiraterone was associated with increased incidence of high-grade anemia. Clinicians should consider the potential adverse events linked to these interventions in the context of clinical practice to enhance patients’ health-related quality of life.

## Data availability statement

The original contributions presented in the study are included in the article/**Supplementary Material**. Further inquiries can be directed to the corresponding authors.

## Ethics statement

The studies involving human participants were reviewed and approved by Ethics Committee of the Ethics Committee of Longhua Hospital, Shanghai University of Traditional Chinese Medicine. The patients/participants provided their written informed consent for the publication in this study.

## Author contributions

ZL: Writing – original draft, Writing – review & editing. BZ: Data curation, Writing – original draft. HX: Project administration, Writing – original draft, Writing – review & editing. LC: Data curation, Software, Writing – review & editing. XS: Formal Analysis, Methodology, Writing – review & editing. YW: Formal Analysis, Validation, Writing – review & editing. RW: Writing – review & editing, Formal Analysis. JZ: Writing – review & editing, Methodology, Validation. YQ: Supervision, Formal Analysis, Resources, Writing – original draft, Writing – review & editing. JY: Writing – review & editing, Validation. YS: Writing – review & editing, Funding acquisition, Supervision, Writing – original draft
