# Supplementary material for: Efficacy and safety of olaparib combined with abiraterone in patients with metastatic castration-resistant prostate cancer: a systematic review and meta-analysis of randomized controlled trials
Source: Front Oncol. 2023 Oct 6;13:1265276. doi: 10.3389/fonc.2023.1265276 (PMC10587563; doi:10.3389/fonc.2023.1265276)
Supplement: Supplementary file 1 [file Table_1.docx]

**Supplementary Table 1. Search Strategy**

**Search Strategy**

Search included: PubMed, Embase, Cochrane Library: until April 27, 2023

Text S1 Search strategy

**Database: Pubmed**

**Search Strategy:**

--------------------------------------------------------------------------------

***Olaparib terms:***

1 " Olaparib"[Mesh]

2 (Olaparib* or AZD-2281* or AZD2281* or AZD 2281* or KU-0059436* or KU0059436*) [Title/Abstract]

3 or/1-2

***Prostatic neoplasms terms:***

4 "Prostatic neoplasms"[Mesh]

5 (Prostate [Title/Abstract]) OR prostatic[Title/Abstract]

6 (Cancer OR tumor OR tumour OR carcinoma OR neoplas* OR malignan* OR phyma) [Title/Abstract]

7 5 AND 6

8 or/4-7

***Randomized controlled trial terms:***

9 "Randomized controlled trial"[Publication Type] or "controlled clinical trial"[Publication Type]

10 (randomized controlled trial* or controlled clinical trial* or randomized* or placebo* or drug therapy* or randomly* or trial* or groups*) [Title/Abstract]

11 or/9-10

***Final search results: Olaparib terms and Prostatic neoplasms terms and Randomized controlled trial terms:***

12 3 and 8 and 11 (101)

Text S2 Search strategy

**Database: EMBASE**

**Search Strategy:**

--------------------------------------------------------------------------------

***Olaparib terms:***

1 'Olaparib'/exp

2 (Olaparib* OR AZD-2281* OR AZD2281* OR AZD 2281* OR KU-0059436* OR KU0059436*):ab,ti

3 OR/1-2

***Prostatic neoplasms terms:***

4 'Prostatic neoplasms'/exp

5 (‘Prostate cancer*’ OR ‘prostate tumOR*’ OR ‘prostate tumour*’ OR ‘prostate carcinoma*’ OR ‘prostate neoplas*’ OR ‘prostate malignan*’ OR ‘prostate phyma*’ OR ‘prostatic cancer*’ OR ‘prostatic tumOR*’ OR ‘prostatic tumour*’ OR ‘prostatic carcinoma*’ OR ‘prostatic neoplas*’ OR ‘prostatic malignan*’ OR ‘prostatic phyma*’):ab,ti

6 OR/4-5

***Randomized controlled trial terms:***

7 'Randomized controlled trial'/exp

8 (‘randomized controlled trial*’ OR ‘controlled clinical trial*’ OR randomized* OR placebo* OR ‘drug therapy*’ OR randomly* OR trial* OR groups*):ab,ti

9 OR/7-8

***Final search results: Olaparib terms and Prostatic neoplasms terms and Randomized controlled trial terms:***

10 3 AND 6 AND 9 (383)

Text S3 Search strategy

**Database: Cochrane Library**

**Search Strategy:**

--------------------------------------------------------------------------------

***Olaparib terms:***

1 MeSH descriptor: [Olaparib] explode all trees

2 (Olaparib* OR AZD-2281* OR AZD2281* OR AZD 2281* OR KU-0059436* OR KU0059436*):ti,ab,kw (word variations have been searched)

3 or/1-2

***Prostatic neoplasms terms:***

4 MeSH descriptor: [Prostatic neoplasms] explode all trees

5 (‘Prostate cancer*’ OR ‘prostate tumOR*’ OR ‘prostate tumour*’ OR ‘prostate carcinoma*’ OR ‘prostate neoplas*’ OR ‘prostate malignan*’ OR ‘prostate phyma*’ OR ‘prostatic cancer*’ OR ‘prostatic tumOR*’ OR ‘prostatic tumour*’ OR ‘prostatic carcinoma*’ OR ‘prostatic neoplas*’ OR ‘prostatic malignan*’ OR ‘prostatic phyma*’):ti,ab,kw (word variations have been searched)

6 or/4-5

***Randomized controlled trial terms:***

7 "Randomized controlled trial"[Publication Type] or "controlled clinical trial"[Publication Type]

8 (randomized controlled trial* or controlled clinical trial* or randomized* or placebo* or drug therapy* or randomly* or trial* or groups*):ti,ab,kw (Word variations have been searched)

9 or/7-8

***Final search results: Olaparib terms and Prostatic neoplasms terms and Randomized controlled trial terms:***

13 3 and 6 and 9 (119)
