# Supplementary material for: Efficacy and safety of olaparib combined with abiraterone in patients with metastatic castration-resistant prostate cancer: a systematic review and meta-analysis of randomized controlled trials
Source: Front Oncol. 2023 Oct 6;13:1265276. doi: 10.3389/fonc.2023.1265276 (PMC10587563; doi:10.3389/fonc.2023.1265276)
Supplement: Supplementary file 2 [file Table_2.docx]

**Supplementary Table 2. Characteristics of patients at baseline.**

| Study name | | Clarke 2018 | | | Clarke 2022 | |
| --- | --- | --- | --- | --- | --- | --- |
| Characteristic | Olaparib and Abiraterone  (n=71) | | Placebo and Abiraterone  (n=71) | Olaparib and Abiraterone  (n=399) | | Placebo and Abiraterone  (n=397) |
| Median age at years (range)  randomization, n (%) | 70 (65-75) | | 67 (62-74) | 69 (43-91) | | 70 (46-88) |
| Age between 18 and 65 | 17 (23.9%) | | 22 (31.0%) | 130 (32.6%) | | 97 (24.4%) |
| Age ≥65 years | 54 (76.1%) | | 49 (69.0%) | 269 (67.4%) | | 300 (75.6%) |
| Race |  | |  |  | |  |
| White | 67 (94.4%) | | 67 (94.4%) | 282 (70.7%) | | 275 (69.3%) |
| Asian | 1 (1.4%) | | 0 | 66 (16.5%) | | 72 (18.1%) |
| Black or African-American | 1 (1.4%) | | 1 (1.4%) | 14 (3.5%) | | 11 (2.8%) |
| Other | 2 (2.8%) | | 3 (4.2%) | 37 (9.3%) | | 39 (9.8%) |
| Time from initial diagnosis to randomization (months) | 62 (38-93) | | 48 (32-76) | 33.6 (4-288) | | 39.5 (1-279) |
| ECOG, n (%) |  | |  |  | |  |
| (0) Normal activity | 34 (47.9%) | | 38 (53.5%) | 286 (71.7%) | | 272 (68.5) |
| (1) Restricted activity | 37 (52.1%) | | 31 (43.7%) | 112 (28.1%) | | 124 (31.2%) |
| Unknown | 0 | | 2 (2.8%) | 1 (0.3%) | | 1 (0.3%) |
| Prior treatment |  | |  |  | |  |
| Prior docetaxel treatment | 71 (100%) | | 71 (100%) | 97 (24.3%) | | 98 (24.7%) |
| Prior treatment with NHA | 0 | | 1 (1.4%) | 1 (0.3%) | | 0 |
| Disease site |  | |  |  | |  |
| Bone | 63 (88.7%) | | 60 (84.5%) | 349 (87.5%) | | 339 (85.4%) |
| Soft-tissue | 38 (53.5) | | 38 (53.5%) | 50 (12.5%) | | 58 (14.6%) |
| HRR status |  | |  |  | |  |
| HRR mutation | 11 (15.5%) | | 10 (14.1%) | 111 (27.8%) | | 115 (29.0%) |
| Wide-type HRR | 15 (21.1%) | | 20 (28.2%) | 279 (69.9%) | | 273 (68.8%) |
| HRR mutation unknown | 45 (63.4%) | | 41 (57.7%) | 9 (2.3%) | | 9 (2.3%) |
| BRCA mutation prevalence |  | |  |  | |  |
| BRCA1 | 0 | | 0 | 9 (2.3%) | | 3 (0.8%) |
| BRCA2 | 2 (2.8) | | 4 (5.6%) | 38 (9.5%) | | 35 (8.8%) |
| PSA concentration (μg/L) | 86 (23-194) | | 47 (21-199) | 17.90 (6.09-67.00) | | 16.81 (6.26-53.30) |
